# Action Intentions, Predictive Processing, and Mind Reading: Turning Goalkeepers Into Penalty Killers

**DOI:** 10.3389/fnhum.2021.789817

**Published:** 2022-01-20

**Authors:** K. Richard Ridderinkhof, Lukas Snoek, Geert Savelsbergh, Janna Cousijn, A. Dilene van Campen

**Affiliations:** ^1^Department of Psychology, University of Amsterdam, Amsterdam, Netherlands; ^2^Department of Human Movement Sciences, Free University of Amsterdam, Amsterdam, Netherlands; ^3^Department of Psychology, Education and Child Studies, Erasmus University Rotterdam, Rotterdam, Netherlands; ^4^Nederlandse organisatie voor gezondheidsonderzoek en zorginnovatie ZonMw, The Hague, Netherlands

**Keywords:** action intention, predictive processing, mind reading, body language, goalkeeper or goalie

## Abstract

The key to action control is one’s ability to adequately predict the consequences of one’s actions. Predictive processing theories assume that forward models enable rapid “preplay” to assess the match between predicted and intended action effects. Here we propose the novel hypothesis that “reading” another’s action intentions requires a rich forward model of that agent’s action. Such a forward model can be obtained and enriched through learning by either practice or simulation. Based on this notion, we ran a series of studies on soccer goalkeepers and novices, who predicted the intended direction of penalties being kicked at them in a computerized penalty-reading task. In line with hypotheses, extensive practice in penalty *kicking* improved performance in penalty reading among goalkeepers who had extensive prior experience in penalty blocking but not in penalty kicking. A robust benefit in penalty reading did not result from practice in kinesthetic motor *imagery* of penalty kicking in novice participants. To test whether goalkeepers actually use such penalty-kicking imagery in penalty reading, we trained a machine-learning classifier on multivariate fMRI activity patterns to distinguish motor-imagery-related from attention-related strategies during a penalty-imagery training task. We then applied that classifier to fMRI data related to a separate penalty-reading task and showed that 2/3 of all correctly read penalty kicks were classified as engaging the motor-imagery circuit rather than merely the attention circuit. This study provides initial evidence that, in order to read our opponent’s action intention, it helps to observe their action kinematics, and use our own forward model to predict the sensory consequences of “our” penalty kick if we were to produce these action kinematics ourselves. In sum, it takes practice as a penalty kicker to become a penalty killer.

## Introduction

Action control has two faces. Not only do we need to coordinate perception and action in order to pursue our motives and accomplish our goals: we also need to coordinate our actions with those of others. Key to the latter is the ability to “read” the actions of others and the intentions behind them. We derive from predictive processing theory the notion that in order to read someone else’s action intention, one needs to have a rich kinesthetic experience with that action oneself (kinesthetic experience refers to what a movement feels like in your own body). In a series of studies, we test the novel and nontrivial hypothesis that penalty-reading performance in soccer improves after practice in penalty-*kicking*.

In a first experiment, we test the hypothesis that the more kinesthetic experience a goalkeeper has in penalty-*kicking*, the more effectively s/he can predict the shooter’s aim, thus improving her/his chances to prevent the shooter from scoring a goal. A second experiment tests the hypothesis that similar benefits can be obtained by motor imagery, that is, by vividly mimicking and experiencing the shooter’s movement in one’s mind. In a third experiment, we train a machine-learning pattern classifier on fMRI data to test (using cross-classification) whether motor-imagery brain networks are engaged in successful penalty reading.

### Penalty Killers

Soccer is one of the most popular sports world-wide. When the stakes are high, such as in knock-out games in the UEFA Champions League or the FIFA World Championship tournament, penalty shout-outs are decisive in 25% of major tournaments matches (Jordet et al., 2007). A fast and well-aimed penalty kick almost never fails. More often than not, however, the results are driven by penalty-shooters who choke under the pressure, or by goalkeepers who distinguish themselves as penalty killers.

Penalty-blocking skills involve both speed and accuracy, which engage in a trade-off: the later the goalkeeper initiates her/his dive, the more information s/he can process about the movement of the shooter and the speed and trajectory of the ball. Hence, the longer s/he waits, the greater the likelihood that s/he will choose the correct direction, but also the greater the likelihood that s/he will be too late. As it turns out, goalkeepers who excel at penalty blocking tend to wait long (Memmert et al., 2013).

Once the shooter has hit the ball, the goalkeeper can use information about the ball’s speed, direction, and rotation to predict at 98% accuracy where it will land. However, waiting and then responding is barely an option: the time it takes for a well-hit ball to cross the goal line and the time it takes for the goalkeeper to respond and arrive are on average close to equal (~600 ms; Franks and Harvey, 1997). Responding after the ball has been hit literally leaves the goalkeeper with too little time to arrive before the ball crosses the goal line (Glencross and Cibich, 1977; Chiappori et al., 2002). Thus, by the nature of the game, goalkeepers are not terribly successful at penalty blocking. In the German *Bundesliga*, they block 18.8% of all penalty kicks (Dohmen, 2008).

Goalkeepers may focus on training reaction speed, but the gain is only marginal. Instead, or in addition, goalkeepers may try and push their luck. One way to do so is by guessing: a risky decision ahead of time to dive left, or right, or to stay in the center, regardless of the shooter’s action (Bar-Eli et al., 2007). Another, more informed way is to use intel about shooter statistics: many penalty shooters have a “favorite angle”, and goalkeepers who have access to this information may take their chances by betting on it. Or else they may *pretend* to know the kicker’s favorite angle, and thus try and intimidate their opponent (who is probably already quite nervous). Other psychological tricks that goalkeepers often entertain include making oneself as tall as possible, trying to engage their opponent in a staring game, distracting them by stalling, by objecting to the ball position, by moving their arms up and down during the pre-shot duration, or by positioning slightly off-center, thus tempting the shooter to aim for the “open” side (Masters et al., 2007; Wood and Wilson, 2010; Weigelt et al., 2012; Memmert et al., 2020).

### “Reading” The Body Language of Penalty Kicks

Alternative, more cognitive ways to improve penalty-blocking success involve attempting to “read” the penalty: assessing the shooter’s kinematic body and movement parameters to predict the direction and speed of the kick (Savelsbergh et al., 2002; Williams, 2009; Dicks et al., 2010; Piras and Vickers, 2011). Penalty-reading skills may well be trainable, and hence of great interest to goalkeepers, coaches, and researchers alike. Thus, goalkeepers should learn to acquire as much information as possible from the run-up and kicking movement of the penalty-shooter in order to improve their blocking performance (Dicks et al., 2010; Memmert et al., 2013).

Optimal visual search helps promote penalty-saving success by having the goalkeeper focus on the speed and direction of the run-up, the position, and orientation of the supporting leg and foot, or the orientation of the torso of the shooter (Savelsbergh et al., 2002; Van Kampen, 2010). Already 100 ms before the shooter hits the ball, these combined kinematic properties are ~85% informative about the direction of the immanent kick. The supporting leg is positioned approximately 250 ms before ball contact, and its orientation is by itself about 80% informative (Franks and Harvey, 1997; Savelsbergh et al., 2002). Experts not only are faster at detecting the relevant information for efficient perception-action coordination (Savelsbergh et al., 2002; Yarrow et al., 2009); they also tend to focus more selectively on the legs, whereas novices also inspect hips, torso, and even arms (Memmert et al., 2013). A proper and timely focus can be learned through training and can help improve penalty-blocking performance by giving the goalkeeper a head start (Savelsbergh et al., 2010b).

Here we go off the beaten path in studying alternative ways of reading the body language of penalty kicks. We will focus on the possibility to *simulate* the observed kinematics as if we engage in that action ourselves, in order to *forward-model the anticipated effects* of “our” action, and then use that *to infer the motive* of the other agent’s action: the intended direction of the penalty kick (“if I were moving like this, then I”d intend to kick the ball in the left lower corner”). Forward modeling is key to modern theories of active inference and predictive processing (e.g., Wolpert et al., 2003; Friston et al., 2011; Clark, 2013), and will be discussed in more detail below.

Darts players can predict where a dart will land on a dartboard by studying the movement kinematics of the thrower, the more accurately so as they are more experienced themselves (Knoblich and Flach, 2001). Likewise, professional basketball players are more accurate at predicting whether a shot at the basket goes in or out (when watching videos that stop at the time the ball is released) than professional coaches, commentators, and journalists (Aglioti et al., 2008). While all of them presumably have similarly extensive experiences in watching such shots, only the players have extensive hands-on experience and hence rich forward models of kicking.

di Pellegrino et al. (1992) observed that so-called mirror neurons fire both when executing a deliberate action and when observing that same action. Mirror neurons help interpret and understand the actions of another individual but also help prime the motor system for one’s own incipient action (Iacoboni et al., 2005; Costantini et al., 2011). Seeing other people’s body movements unconsciously activates motor representations in the observer’s brain (Fadiga et al., 1995; Rizzolatti et al., 2009). This so-called motor resonance (Gallese, 2001) suggests that individuals subconsciously simulate someone else’s action. Note that the activity of the motor system is not *exactly* identical between observing and executing an action—if this were the case, then a person would move every time they observed another person acting (Babiloni et al., 2016, 2017). Brain regions rich in mirror neurons show increased activation when anticipating the opponent’s movements in soccer (Bishop et al., 2013).

Mirroring a movement is not always adequate, however: if someone hands you a coffee mug by holding its ear, one needs to *complement* rather than mirror the other’s action (Sebanz et al., 2006; Sartori et al., 2012). An observed action must first be read and comprehended in order to infer its intent (s/he aims to hand me the coffee); next, the observed action should be linked to appropriate complementary actions (to grasp the mug I should open my hand, as s/he holds it by the ear) (Sartori and Betti, 2015). In such situations, unconscious motor resonance reflects not only the imitative kinematics of the observed actions but also the predicted kinesthetic effects of our response (Sartori et al., 2015).

Penalty situations likewise entail complementary actions. The goalkeeper needs to infer, based on observations of the kicker’s run-up and shooting kinematics, the orientation of the supporting leg, etc.), the intention of the shooter (which angle will s/he take), and then act accordingly. Generalizing from the darts and basketball examples, we may argue that for goalkeepers to read the body language of penalty kicks, they should be experts in kicking penalties themselves—an entirely novel conjecture.

### Predictive Processing

Crucial to the theory’s credit (and wider applicability in elite and amateur sports) will be an empirical demonstration not only of the predicted effect but also of the neurocognitive mechanisms through which reading the shooter’s actions promote successful penalty-blocking. Compatible with the darts and basketball findings, a view on reading others’ action intentions in terms of predictive processing was proposed by Ridderinkhof and Brass (2015). These authors derived predictions about penalty-blocking skills from a specific instantiation of predictive processing theory, the *Impetus, Motivation, and Prediction in Perception-Action Coordination Theory* (IMPPACT; Ridderinkhof, 2014).

Predictive processing theories such as IMPPACT refer to the brain metaphorically as a “prediction pump”, constantly predicting the effects of one’s actions in order to optimize the selection of actions appropriate for obtaining present goals. Such predictions are made using forward models: rapid computational algorithms that predict the consequences of one’s actions (as perceived through exteroceptive senses, such as our eyes; or through interoceptive senses, such as proprioception: “how does the movement feel”). The forward model stores the link between the specific kinematic parameters of the action, the specific kinesthetic experience associated with that action, and the specific effects of that action in the world. Information stored in forward models emanates from prediction errors, which arise from the discrepancy between the *desired* consequences of one’s action on the one hand, and either the *observed* or the *predicted* consequences on the other. By testing model predictions and minimizing prediction errors, the forward model becomes more and more accurate. By practicing or simulating the action over and over, in a variety of circumstances, one’s forward model is gradually augmented, so that it provides an increasingly rich and accurate repertoire in predicting the consequences of one’s movements (Wolpert et al., 2003) across a variety of situations—such as in penalty-kicking and penalty-blocking.

In movement-reading, we apply forward models to predict the consequences of actions executed by others rather than oneself. The richer one’s forward model, the better one will be able to “inverse model”, and hence predict, the effects of the corresponding action executed by someone else (Kilner et al., 2007; Ridderinkhof, 2017). A rich forward model is built on extensive kinesthetic experience; thus, for goalkeepers aiming to block a penalty, IMPPACT suggests that the more kinesthetic experience a goalkeeper has in penalty-*kicking*, the more effectively s/he can inverse model and predict the shooter’s aim, thus improving her/his chances to prevent the shooter from scoring a goal (Ridderinkhof and Brass, 2015). Here we will test this proposal empirically, in a sample of experienced goalkeepers.

### Pre-Play: Kinesthetic Motor Imagery

The notion of forward modeling of the proprioceptive consequences of one’s action bears resemblance to the notions of *kinesthetic motor imagery* (KMI). In KMI, one performs and experiences a movement in one’s mind, vividly but without moving (Moran et al., 2012). One pre-plays the movement, as it were. Practicing through mental pre-play can help improve a movement (and hence learning to perform it optimally; Ziessler and Nattkemper, 2002; Ridderinkhof, 2014). KMI engages a first-person perspective (rather than the third-person perspective in visual motor imagery): an act is “seen” through the person’s own eyes and “felt” through the person’s own interoceptive senses. Gymnastic athletes report realistic kinesthetic sensations during KMI of a complex gymnastics exercise, the so-called Yurchenko jump (Calmels et al., 2018). We conjecture that the more vivid one’s KMI, the more one’s forward model can gain in precision. The present study will put this further proposal to the test by giving participants experience in observing and pre-playing penalties to see if their penalty-reading skill improves.

The notion that kinesthetic experience can be acquired through KMI relies on the assumption of functional equivalence: physical movements and their mental (imagined) counterpart engage similar neural circuits and neurophysiological processes (Decety and Jeannerod, 1996), and hence largely activate the same brain areas (Ridderinkhof and Brass, 2015). Neural activation during KMI resembles the preparatory planning phase that precedes movement (Jeannerod, 2006), but also goes beyond mere preparatory planning, as demonstrated by the finding that KMI engendered activation in the contralateral primary motor cortex just as actual movements did (Stinear et al., 2006).

**Figure 1:**
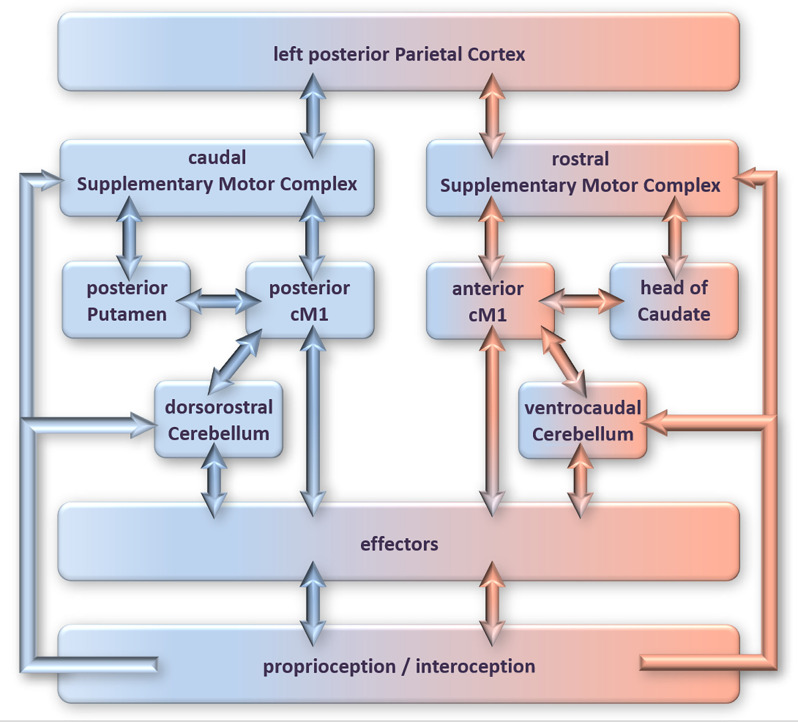
The neural circuitry involved in overt motor performance (blue/left side of figure) compared to motor imagery (rose/right side of figure/nodes). Adopted from Ridderinkhof and Brass (2015); for details please refer to that article.

fMRI and lesion studies have produced a fair picture of the network of brain regions recruited by actual movement execution and mental pre-play of the same movement (an overview of these networks is depicted in Figure 1; details are beyond the present scope). As reviewed in Ridderinkhof and Brass (2015), these networks largely overlap, with the differences between play and pre-play characterized by spatial gradients (visualized in Figure 1) rather than the recruitment of entirely different regions (for the details, which go beyond the present scope, we refer the reader to our previous review).

Multivariate pattern analysis (MVPA) is an increasingly popular analysis technique to quantify the involvement of cortical networks in particular perceptual or cognitive processes. Here, we use MVPA of fMRI data to test whether the individual motor-imagery brain networks are engaged in successful penalty reading. We first train a classifier to discern, separately for each individual, the circuitry unique to motor imagery from the circuitry involved in viewing penalties for individual participants in general. Subsequently, we test if individuals use their motor imagery circuitry to successfully read penalty kicks and predict their direction. This will be the aim of the third experiment reported here.

## Experiment 1: Practicing Penalty Kicking

Deriving from a set of assumptions embodied in the IMPPACT theory, here we test the hypothesis that goalkeepers, in order to improve their penalty-reading skill and hence their penalty-blocking performance, should develop kinematic and kinesthetic experience in penalty-*kicking*.

In a sample of experienced goalkeepers, playing in high-level amateur competitions, we create three groups: one group of goalkeepers who practice in penalty-kicking; another who practice in penalty-blocking (in conventional ways); and finally a control group of goalkeepers who practice in non-penalty-related soccer skills, under otherwise comparable circumstances. The main aim of having the control group is to establish the baseline improvement from pre-test to post-test in performance in the penalty-reading task. Beyond such practice effects (or their counterparts: effects of fatigue or boredom), such a control group also helps rule out interpretations of training improvements in terms of the effects of motivation, attention, expectation, and the like.

Goalkeepers who practice penalties should improve more than those in the control group. Goalkeepers in the penalty-blocking group may obviously improve because of training penalty-blocking itself (building on their prior experience). Goalkeepers in the penalty-kicking group may improve because the enrichment of their forward model of penalty kicking will allow a more optimal reading of the body language and intention of the penalty shooter. The latter prediction is, to our knowledge, unique to IMPPACT (although other varieties of predictive processing theory can readily be extended to include such assumptions). Since the penalty-blocking group builds on prior experience and hence had less room for improvement, we might expect the penalty-kicking group to improve most. Nonetheless, any observation of improvement in the penalty-kicking group (compared to the control group) would already satisfy our theoretical prediction.

Conceivably, individuals with greater interoceptive awareness of their bodily senses (Khalsa and Lapidus, 2016) may benefit from the richer kinesthetic experience and hence more effective forward and inverse modeling. Thus, we include an interoceptive awareness scale to examine whether higher scores come with greater penalty-reading success.

### Methods

#### Participants

Goalkeepers were recruited via high-level amateur soccer clubs and goalkeeper training centers in the Netherlands. Inclusion criteria were an age of 16 or older and a minimum of 3 years of active experience as a goalkeeper in an amateur or (semi-) professional soccer team participating in a competition of the Royal Dutch Soccer Association (KNVB) in the period immediately preceding the experiment. Exclusion criteria were more-than-minimal prior experience in kicking penalties (i.e., at least monthly during the past year). Our remaining sample consisted of 51 male goalkeepers with a mean age of 22.8 years (range 16–60 years). Participants could win one of five vouchers of 25€ each assigned through a lottery in return for their participation. They provided informed consent before participation. All procedures were approved by the university ERB (nr. 2017-DP-8029) and complied with relevant laws and institutional guidelines.

#### Design

Using a pre-test/post-test non-equivalent group design, participants were assigned pseudo-randomly to three groups, with the restriction that goalkeepers from the same soccer club or training center formed duo’s in two of the groups (see below). In the penalty-blocker (PB) group, goalkeepers practiced blocking penalties in the conventional way (as detailed further under *Procedures*). In the penalty-shooter (PK) group, goalkeepers practiced *kicking* penalties instead of blocking them. In the control (C) group, participants ran a series of 80 meters, and practiced keeping the ball in the air (using all body parts except their arms and hands, and without holding the ball in any way), for as long as possible.

In the PB and PK groups, goalkeepers were matched in pairs. They performed individually but formed duo’s to facilitate procedures. One of the two goalkeepers always kicked the penalty shots (and never blocked them, consistently throughout the experiment), while the other always blocked them (and never kicked them). Goalkeepers were assigned to duo’s pseudo-randomly, such that both goalkeepers in a pair played at the same club and trained together, or at least trained an equal amount of time in the same environment. Matching was based on level of goalkeeping experience, ranking within the team (first or second goalkeeper), and age, as much as possible.

#### Materials

##### Training

During PB and PK training, a standard full-sized soccer goal (7.32 m wide × 2.44 m wide) was used and penalties were shot from the standard distance of 11 meters from the goal. All three groups practiced with a standard size soccer ball on a regular training field, with all participants wearing their typical training gear.

###### Penalty-Reading Task

Goalkeepers performed a computer task twice on a 15-inch laptop to assess penalty-anticipation skills. Video clips showed a soccer player running up to the ball to shoot a penalty from the 11-meters dot on a regular soccer pitch (kickers were youth players of PSV Eindhoven Football Club; materials were adapted from Savelsbergh et al., 2010a). Penalties were videotaped from the goal-line, thus rendering a goalkeeper’s first-person perspective. At the moment the shooter’s foot touched the ball, the clip was arrested, and (with a fade-out time of 0.5 s) the screen turned green (the same color as the grass of the soccer pitch). The time between the start and “arrest” of the clip varied between 1.2 and 1.5 s. Participants were asked to predict the direction of the ball by pushing one of four buttons, arranged in a spatially compatible fashion. Each button (keyboard letters E/I/C/N) referred to one of four possible sections in the goal shown on the screen, divided into right/left, and low/high. Thirty unique video clips, with 15 different penalty kickers, were shown in randomized order. Each test consisted of six practice trials and 60 test trials. Instructions emphasized accuracy, but also stressed speed of responding (since in real-life penalty-blocking, diving in the correct direction won’t save a goal if the dive is too late). Accuracy is defined as the percentage of responses in which the selected button corresponded with the actual penalty direction “behind the video”. Reaction time was measured at each trial as the interval (in milliseconds) between the moment the shooter touched the ball and the moment the participant hit the button. After every block of trials, accuracy was displayed on the screen. *Presentation* software was used to show the video clips, record the responses, and control the experiment. The task was based on materials and prior experience with a similar task in previous research (Savelsbergh et al., 2002).

###### Penalty-Kicking Experience Questionnaire

Participants filled out a questionnaire that asked for goalkeeper experience (0–1 year, 1–3 years, 3–5 years, >5 years), experience in penalty kicking (yes/no), and if yes, at which frequency (a few times a year, every month, every 2 weeks, or every week).

###### Interoceptive Awareness Questionnaire

Participants also filled out a brief self-developed questionnaire on interoceptive awareness. They rated 10 statements on a 7-point Likert scale from never to always. Example statements are “when I move, I can focus my attention on how that movement feels physically”, “when I see someone moving, I can feel in my own body how that movement feels”, and “I notice changes in my body, such as breathing faster or slower, or a change in my heart rate”. These statements were selected and compiled from existing questionnaires on interoception; our selection has not been validated or tested for reliability as such.

#### Procedure

The experimenters visited the goalkeepers at their clubs/training centers. After a brief introduction, the goalkeepers were seated individually in a silent room, where they first provided informed consent. They filled out the questionnaires and then were administered the penalty-reading task as a pre-test. This part of the session took ~25 min, including ~15 min for the penalty-reading task. Participants were then provided with standardized instructions on the training to be carried out. The instruction emphasized taking their time, and focusing on how it feels to kick/block a penalty or how the movements to keep the ball in the air feel. In the PK/PB groups, the goalkeeper duo’s kicked/blocked penalties in the conventional way. They were instructed to take a moment before each penalty to plan their kick/save, and after each kick/save to recall how well it went and, especially, how it felt in their body. After a series of 10 kicks/saves there was a 1-min break. The training entailed four series of 10 penalties and lasted ~20 min in total. Goalkeepers in the control group were instructed to run 80 meters, fast but not at full sprint speed, and then try and keep the ball in the air as long as possible during 1 min. After a 1-min break, the next series of 80 meters running and 1 min of keep-the-ball-in-the-air started. There were four series, lasting ~20 min in total. To stir up motivation, participants in each group kept article records of their performance during each 1-min break (these data were not analyzed). After the training, the penalty-reading task was administered again as a post-test. Finally, the goalkeepers were debriefed. All procedures involved the continuous presence of an experimenter. The entire session lasted approximately 1 h.

#### Statistical Analysis

In the data obtained from the penalty-reading task, trials where participants gave no response at all were discarded from analysis (<1%). Reaction times could in principle be negative since participants were encouraged to respond as soon as they thought they knew the direction of the kick; sometimes this was already before the ball was kicked, which in theory is possible (indeed, goalkeepers don’t always wait).

Accuracy scores and average reaction times were analyzed (separately) using analysis of variance (ANOVA), with interoceptive awareness as a covariate in a follow-up ANCOVA. Group was entered as a between-subjects variable (control, PB, PK) while Time was entered as a within-subjects variable (pre, post). Bayes Factors (BF) were calculated to assess how much more probable the observed data under H_0_ was than under H_A_. That is, we report BF_01_, not BF_10_. In case of a significant interaction, paired-samples *t*-tests were used to examine pre-post differences per group.

### Results

#### Accuracy

**Figure 2:**
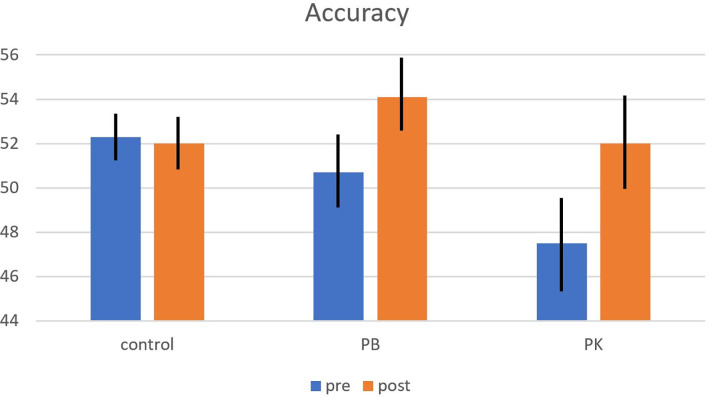
Interaction of the effects of Group (X-axis) and Time (separate bars/violins) on penalty reading accuracy (Y-axis) in a bar graph (left panel) and a violin plot (right panel). Accuracy improved from pre- to post-test for both the PB and PK training groups, but not for the control group. Error bars (left panel) represent 1 standard error to the mean.

Accuracy averaged 51.3%, which is well above chance level (25%), indicating that participants were reasonably well able to predict penalty direction from the video clips. Groups did not differ in penalty-reading accuracy (*F*_(2,48)_ = 1.00, *p* = 0.375, *η*^2^ = 0.040, *BF* = 2.445). Accuracy improved from pre- (50.1%) to post-training (52.5%) tests (*F*_(1,48)_ = 11.20, *p* = 0.002, *η*^2^ = 0.189, *BF* = 0.138). Most important, the effect of training differed between training groups (*F*_(2,48)_ = 3.99, *p* = 0.025, *η*^2^ = 0.143; the BF for the full model of both main effects and the interaction term equalled 0.133, signaling that the probability of these data was considerably lower under H_0_ than under H_A_). As depicted in Figure 2, accuracy improved for both the PB and PK groups, but not for the control group. This interaction pattern survived after partialing out covariance with interoceptive awareness (*F*_(2,48)_ = 4.19, *p* = 0.021, *η*^2^ = 0.151), indicating that it was not produced by group differences in interoceptive skill.

When zooming in *post hoc* on pre-post differences per group, we observed that both the PB and PK groups improved from pre- to post-test (*t*_(16)_ = −3.29, *p* = 0.005; and *t*_(14)_ = −3.21, *p* = 0.006, respectively), whereas the control group did not (*t*_(18)_ = 0.25, *p* = 0.803). The improvement in accuracy was numerically greater for the PK than the PB group (4.5 vs. 3.4%, but this difference did not obtain statistical significance in an independent-samples *t*-test (*t*_(30)_ = −0.69, *p* = 0.498).

#### Response Speed

Response speed averaged 664 ms. Groups did not differ in penalty-reading speed (*F*_(2,49)_ = 1.93, *p* = 0.156, *η*^2^ = 0.073, *BF* = 1.238). Reaction time improved from pre- (691 ms) to post-training (636 ms) tests (*F*_(1,49)_ = 4.81, *p* = 0.033, *η*^2^ = 0.089, *BF* = 1.858). Most important, the effect of training did not differ between training groups (*F*_(2,49)_ = 1.49, *p* = 0.235, *η*^2^ = 0.057; the BF for the full model of both main effects and the interaction term equalled 1.135, signaling that the probability of these data was considerably higher under H_0_ than under H_A_). This interaction pattern was not altered after partialing out covariance with interoceptive awareness (*F*_(2,49)_ = 1.66, *p* = 0.201, *η*^2^ = 0.065).

### Discussion

Practicing in penalties improved penalty-reading accuracy. Not surprisingly, this held for practicing in penalty-blocking: practicing one’s physical penalty-blocking skill could well be expected to generalize to improved penalty-reading skill in “virtual space”, even though the former includes features that are not entailed in the latter (such as the actual dive, which can fail even if it is in the correct direction, and which feels more real).

What is less trivial, and confirms our hypothesis, is that practicing in penalty-*kicking* improved penalty-reading accuracy as well. This can clearly not be explained by experiencing penalty-blocking situations *per se*. It might potentially be explained by increased experience in the specific penalty-reading task that we used, but this account fails to receive support by the finding that penalty-reading accuracy did *not* increase in the control group. A remaining difference between control and penalty groups is that the latter underwent “general practice” in penalty situations, which might benefit their penalty-reading skill even for goalkeepers who practiced kicking rather than blocking. However, we fail to see a possible mechanism behind such an effect, especially given that goalkeepers in the control and PK conditions alike already have quite abundant experience with penalty situations in general. Thus, we conclude that the hypothesis derived from the IMPPACT model in terms of forward modeling is much more specific and at this point cannot be discarded.

The improvement in accuracy in the PK group was 4.5%, which may seem small, but is meaningful nonetheless: a small increase in penalty-reading accuracy (after merely 20 min of training) may well instantiate the difference between winning and losing a match.

Obviously, small sample sizes are always a hazard, as they are here. For example, it cannot be excluded that the finding that goalkeepers in the PK group scored relatively low at pre-test might be a consequence of sampling error. Pre-test accuracy differed between groups, ranging from 43.3–60.0% in the control group to 40.0–59.2% in the PB group and 33.3–59.2% in the PK group. The latter group scored lowest at pre-test and improved the most. However, when, by way of an exploratory analysis, we remove the only two participants who scored below 40% at pre-test (both scoring 33.3%, both in the PK group) from the analysis, the patterns remain very much the same, with the interaction effect remaining highly comparable both qualitatively as quantitatively, even though the accuracy at pre-test has now become quite similar across groups. Thus, this specific alternative account in terms of sample error does not seem to be supported by the data, although obviously, the sample size remains small, and the present finding stands to await independent replication.

Practicing exerted a considerable improvement in penalty-reading speed (55 ms) in the same direction as it did for accuracy; however, this effect was not modulated by training condition, and hence cannot be attributed specifically to forward modeling or any other specific factor. Although practice in the penalty-reading task did not improve accuracy in the control group, it did improve speed, so it would seem that this was a mere practice effect.

The observed patterns of findings were not modulated by interoceptive awareness scores, which ran counter to our intuition. Our instrument to measure interoceptive awareness was based on existing instruments, but it was brief and selective, and at any rate, not yet validated. Thus, future studies may aim to replicate the present findings with more optimal measures of interoceptive awareness.

Our sample was relatively homogeneous in terms of goalkeeping experience. Future studies might include players that differ in level and years of experience to see if these moderate the observed effects.

In conclusion, this experiment provides initial evidence that practicing penalty-kicking improves penalty-reading accuracy. The assumption that the improvement in penalty-reading skill, as ensuing from enriched forward models of penalty-kicking, actually involves pre-play was tested in the next experiment.

## Experiment 2: Kinesthetic Motor Imagery of Penalty Kicking

The notion of forward modeling of the proprioceptive consequences of one’s action is thought to invoke kinesthetic motor imagery or rapid pre-play. It has been argued and shown that mental action simulation alone can aid in learning a specific motor skill (Ziessler and Nattkemper, 2002; Ziessler et al., 2004). Here we assess the power of pre-play by giving participants experience in observing and pre-playing penalties to see if their penalty-reading skill improves.

A sample of novices was divided into two groups: one group that practices in KMI of penalty-kicking; and one that practices in KMI of penalty-blocking. The groups are exactly comparable in all other respects, including materials and instructions other than the focus on penalty kickers and goalkeepers. A separate control group was omitted since the previous experiment had already demonstrated that mere effects of practice, expectation, and motivation on performance in the penalty-reading task were negligible.

Goalkeepers in the penalty-kicking group may improve because KMI of the movements of the penalty kicker enriches their forward model of penalty kicking, which should allow for more optimal reading of the intention of the penalty kicker. The latter prediction is, again, to our knowledge, unique to IMPPACT. Goalkeepers in the penalty-blocking group may improve as well, either because KMI may enrich their forward model of penalty blocking, which might benefit the speed and accuracy of their response to penalties fired at them, or because of more generic experience with penalty-blocking situations. While we might expect the penalty-kicking group to improve most, observing that this group improved at all would already satisfy our theoretical prediction.

KMI might be likened to empathic perspective-taking, often described as “understanding another’s point of view”. Shared representations in the perception and action of motor behavior correspond to shared representations between understanding and experiencing the state of another (Preston, 2007), presuming a kind of bodily merging of the self with the other (Erle and Topolinski, 2015). Conceivably, then, individuals with higher dispositional empathy may be more proficient at KMI and at action-intention reading. Thus, we include an empathy scale to examine whether higher scores come with greater penalty-reading success.

### Methods

#### Participants

Participants were first-year Psychology students at the University of Amsterdam who participated in return for course credits. They ranged in age from 18 to 35 years. Exclusion criteria were prior experience as a field player or goalkeeper in a regular soccer team, either anytime in the past 2 years, or for a total of more than 1 year in the farther past. Our remaining sample consisted of 55 participants (33 females) with a mean age of 21, 7 years (range 18–29 years). They provided informed consent before participation. All procedures were approved by the university ERB (nr. 2017-DP-7945) and complied with relevant laws and institutional guidelines.

#### Design

Using a pre-test post-test non-equivalent group design, participants were assigned pseudo-randomly to one of two groups, with the restriction that groups of up to four participants who participated simultaneously were always assigned to one and the same group, so as to prevent them from entertaining hypotheses about which of the training regimes might work best. Participants in the penalty-blocker (PB) group practiced KMI of blocking penalties, while participants in the penalty-kicker (PK) group practiced KMI of *kicking* penalties (as detailed further under *Procedures*).

#### Materials

##### Penalty-Reading Task

This task was identical to the one described under Experiment 1.

##### Penalty-Kicking Experience Questionnaire

Participants filled out a questionnaire that asked for soccer field player and goalkeeper experience (0–1 year, 1–3 years, 3–5 years, >5 years), experience in penalty kicking (yes/no), and if yes, at which frequency (a few times a year, every month, every 2 weeks, or every week).

##### Empathy Questionnaire

Participants also filled out the Dutch translation of the *Basic Empathy Scale* (Jolliffe and Farrington, 2006; Van Langen et al., unpublished manuscript). They rated 20 statements on a 5-point Likert scale from “entirely disagreed” to “entirely agreed”. Example statements are “I feel sad when I see people cry, ” “I get carried away easily by the feelings of others, ” and “I have a hard time grasping when my friends are happy.” Reliability coefficients for the subscales ranged from *α* = 0.72–0.81.

##### KMI Training Materials

During the training, participants watched a series of 75 unique video clips, each showing a penalty kick. The video clips show penalties that have taken place in actual matches and feature professional soccer players. Fragments were clipped from YouTube footage of penalty shoot-outs from European Champions League matches or from UEFA European Championship or FIFA World Championship tournament matches. Each clip was filmed from behind the penalty shooter, such that both the shooter, the goalkeeper, and the goal were in full view. The ball was at the 11-meters spot; the goalkeeper was at the goal-line. Each video clip lasted between 1.2 and 1.5 s and was shown twice in succession. Clips were separated by a black screen for 1 s. The duration of one block of clips was 10 min. Participants were instructed to imagine, as lively as possible, the bodily feeling of the movements of the goalkeeper (in the PB group) or of the penalty kicker (in the PK group) during each of the penalties. Informal pilot work had indicated that KMI was much facilitated by the immediate repetition of each fragment.

#### Procedure

The experiment took place in the labs of the University of Amsterdam. Groups of up to four participants were instructed together but performed the tasks in separate cubicles. After a brief general explanation they provided informed consent, and then were administered the penalty-reading task as a pre-test, which took about 15 min. Next, they received instructions for the KMI training, and then took two blocks of the training, separated by a few minutes rest. The training lasted for 21 min. They were then administered the penalty-reading task again, as a post-test, which again took about 15 min. Finally, they filled out the empathy questionnaire, followed by debriefing. The entire session lasted approximately 1 h.

#### Statistical Analysis

In the data obtained from the penalty-reading task, trials where participants gave no response at all were discarded from analysis (<1%).

Accuracy scores and average reaction times were analyzed (separately) using analysis of variance (ANOVA), with empathy scores as a covariate in a follow-up ANCOVA. Group was entered as a between-subjects variable (PB, PK) while Time was entered as a within-subjects variable (pre, post). Bayes Factors (BF) were calculated to assess how much more probable the observed data under H_0_ was than under H_A_.

### Results

Two participants scored at chance level after training (23.3% and 26.7%), whereas all others scored (well) over 30%. Two other participants were excessively slow in responding (>1,500 ms), whereas all others scored 1,138 ms or below. These four participants (all from the PB group) were removed from the sample, although we verified that the results presented below were not influenced qualitatively by their removal.

#### Accuracy

Accuracy averaged 43.8%, which is well above chance level (25%), indicating that participants were reasonably well able to predict penalty direction from the video clips. Groups did not differ in penalty-reading accuracy (*F*_(1,49)_ = 2.87, *p* = 0.097, *η*^2^ = 0.055, *BF* = 1.006). Accuracy improved slightly from pre- (43.5%) to post-training (44.0%) tests, but this effect was not statistically robust (*F*_(1,49)_ = 0.23, *p* = 0.632, *η*^2^ = 0.005, *BF* = 4.406). Most important, the effect of training did not differ between training groups (*F*_(1,49)_ = 0.02, *p* = 0.902, *η*^2^ = 0.000; the BF for the full model of both main effects and the interaction term equalled 14.624, signaling that the probability of these data was far higher under H_0_ than under H_A_). This interaction pattern did not change after partialing out covariance with empathy scores.

#### Response Speed

Response speed averaged 711 ms. Groups did not differ in penalty-reading speed (*F*_(1,49)_ = 0.04, *p* = 0.835, *η*^2^ = 0.001, *BF* = 2.024). Reaction time improved from pre- (723 ms) to post-training (699 ms) tests, but this effect was not statistically robust (*F*_(1,49)_ = 1.22, *p* = 0.274, *η*^2^ = 0.024, *BF* = 2.908). The effect of training id not differ between training groups (*F*_(1,49)_ = 0.28, *p* = 0.596, *η*^2^ = 0.006; the BF for the full model of both main effects and the interaction term equalled 18.166, signaling that the probability of these data was far higher under H_0_ than under H_A_). Partialing out covariance with empathy id not alter this interaction pattern.

### Discussion

Practicing in kinesthetic motor imagery of penalties failed to improve penalty-reading accuracy, in either the penalty-blocking or the penalty-kicking condition, thus disconfirming our predictions. Our KMI sessions appeared to lack the power to produce the improvements in penalty anticipation that we observed after practice in physical penalty kicking in Experiment 1. This finding falsifies part of the predictions derived from the IMPPACT theory: KMI of penalty kicking failed to strengthen and enrich the forward model of penalty kicking enough for the observer to improve in reading an opponent’s penalty kick.

A number of observations may limit this straightforward falsification. First, the fact that practice in KMI of penalty *blocking* also failed to produce improvements may suggest that our KMI sessions were not successful in instilling *any* effects, at least in the present samples. A more successful implementation of KMI might still produce the expected effects; this remains to be tested. Second, and relatedly, the present sample consisted of novices with little experience in penalty kicking or blocking. Stronger effects might be obtained by testing goalkeepers, as in Experiment 1. Note that both accuracy and response speed were considerably poorer among the novices in Experiment 2 than in the goalkeepers in Experiment 1. For instance, experienced players may have a more refined notion of what aspects of movement to imagine. Third, as noted before, sample sizes were relatively small, amplifying the risk of false negative findings. However, our finding was not a case of a sizable effect that failed to reach statistical robustness; rather, in the present samples the effect was just negligible.

The video clips used for KMI training showed the shooter from behind, whereas those used for the penalty-reading task showed the shooter from the front. This difference in viewpoint might limit the effect of training. Yet, we opted for this difference for two reasons. First, if the same point of view were used for both the imagery training and the penalty-reading task, then the two would become more visually similar, such that penalty-reading performance might benefit simply from rehearsing similar material rather than from practice in motor imagery. And second, for the motor-imagery training materials, the movements of both the goalkeeper and the shooter should be in full view, in order that the same clips can be used both for motor imagery of the goalkeeper and for imagery of the shooter; this is difficult to accomplish from behind the goal (and virtually no footage is available from that viewpoint).

Individuals may vary considerably in terms of the vividness of their KMI, both in general (as a dispositional trait) and in the present set-up (as a situational state). Possibly, for some individuals in the present experiment, the vividness of KMI was limited, which would also limit the chances of finding *any* effect of KMI practice. Future studies may incorporate instruments to measure KMI skills.

Vivid KMI may depend in part on prior actual (physical) experience with the skill being practiced (Ridderinkhof, 2014). Thus, perhaps KMI should build on physical training rather than being administered separately. A replication (with larger samples) might focus on *combining* physical and virtual training, and might compare novices to experienced goalkeepers to examine if and when KMI might contribute to performance beyond physical training.

The effects of KMI were not modulated by empathy, which is no surprise given the lack of effect of KMI. Potential relationships between KMI efficacy and empathy remain to be explored further.

The finding that practice in KMI of penalty-kicking and penalty-blocking did not improve penalty-reading skills does not exclude the possibility that KMI *is* used during penalty reading in the first place. This possibility was tested in a further experiment, described next.

## Experiment 3: An MVPA-Based Analysis of Pre-Play in Reading Penalty Kicks

Multi-variate pattern analysis (MVPA) of fMRI data is a technique that can quantify the difference between multivariate patterns of neural activity associated with different classes of cognitive, affective, or behavioral factors (Haxby, 2012). MVPA has become popular because of its sensitivity to slight differences in activity patterns that univariate techniques have more difficulty detecting (Haynes and Rees, 2006). In MVPA, a machine-learning classifier algorithm is trained on data from a subset of the experiment and then tested on the remaining subset of data. During training, the classifier is informed about the condition from which the test trials came, so that it can learn which patterns of activation across voxels distinguish the conditions. Learning is successful if classification performance transfers with greater-than-chance accuracy from the training set to the testing set. Thus, through its ability to “decode” information in the test set, MVPA constitutes a test of the difference between multivariate neural representations (Snoek et al., 2019).

An emerging trend in such machine-learning classifiers is cross-classification, which capitalizes on its power to provide evidence for *similarity* among neural patterns. When a classifier is trained on data from one cognitive task and tested on data from another, conclusions can be drawn about the role of specific clusters of voxels in the brain in cognitive processes that generalize across those two tasks (Kaplan et al., 2015). MVPA cross-classification (MVPA-CC) has proven useful in establishing correspondences among neural patterns across a variety of cognitive domains, including neural overlap between self-focused emotion imagery and other-focused emotion understanding (Oosterwijk et al., 2017) in our lab.

Here we apply MVPA-CC to the question whether the neural circuits engaged in KMI are also engaged in reading the body language of penalty-kicking. We will first train a classifier to discern, separately on half of each individual’s data, the circuits unique to motor imagery from the circuits involved in attention to kinematic features of penalties (Note that such attentive viewing occurs in KMI as well; we are interested in the circuitry that *distinguishes* “pure” KMI circuitry from the more generic circuitry.). We then test the trained classifier on the remaining half the data per individual, to see if classification accuracy is above chance. Subsequently, we will use cross-classification to test if individuals use their motor imagery circuitry to successfully read penalty kicks and predict their direction. We hypothesize that successfully read penalties (in which direction was correctly anticipated) are associated with activation of voxels that were identified in the KMI task.

### Methods

#### Participants

Participants were students at the University of Amsterdam who were rewarded €25 in return for their participation. All participants in the experiment reported no known medical or psychological problems, right-hand dominance, not taking medication, no psychiatric disorders or neurological history, and normal color or corrected-to-normal vision acuity, and no head injury. Exclusion criteria were general MRI contraindication (e.g., claustrophobia, metal implants, possible metal scraps), prior experience as a field player or goalkeeper in a regular soccer team, either anytime in the past 2 years, or for a total of more than 1 year in the farther past; or a self-reported history of neurological or psychiatric disorders. Our remaining sample consisted of 35 participants (19 females) ranging in age between 22 and 38 years. Participants provided informed consent before participation. All procedures were approved by the university ERB (nr. 2016-DP-7251), and complied with relevant laws and institutional guidelines.

#### Materials

The *penalty-kicking experience questionnaire* was identical to the one described under Experiment 1.

The *penalty-reading task* was identical to the one described under Experiment 1, with the exception of timing. Clips were separated by intervals of on average 5 s (2–8 s, jittered in steps of 0.1 s), with a break of on average 5.5 s (3–8 s, jittered in steps of 0.1 s) after every series of 3 clips. These mini-blocks of three jittered trials and a jittered break served to prevent saturation of the BOLD signal.

##### KMI Training Materials

Each video clip lasted between 1.2 and 1.5 s and was shown twice in succession. Clips were separated by a black screen for 1 s. The duration of one block of clips was 10 min. Participants were instructed to imagine, as lively as possible, the bodily feeling of the movements of the goalkeeper (in the PB group) or of the penalty kicker (in the PK group) during each of the penalties. Informal pilot work had indicated that KMI was much facilitated by the immediate repetition of each fragment.

The *video training materials* consisted of the same fragments as described under Experiment 2. Video clips lasted between 1.2 and 1.5 s and were separated by intervals of on average 5 s (2–8 s, jittered in steps of 0.1 s), with a break of on average 5.5 s (3–8 s, jittered in steps of 0.1 s) after every series of three clips. In between clips, rather than a black screen, the goal area was blurred (including the goalkeeper, the penalty kicker, and the advertisement billboard surrounding the goal). All video clips in a block of four mini-blocks were viewed under the same instructions, twice under “attention” instructions (ATT) and twice under “imagery” instructions (IMG) (as described under *Procedures* below). A brief cue indicating the task instruction (“ATTENTION” or “IMAGERY”) was shown during 5 s, followed by the first clip after an interval of 5.5 s (3–8 s, jittered in steps of 0.1 s). The two instructions were given in AABB order to half of the participants, and in BBAA order for the other half, such that participant received two blocks of four mini-blocks each under one instruction, and then two blocks of four mini-blocks each under the other instruction.

#### Procedure

The experiment took place in the labs of the University of Amsterdam Spinoza Center for neuroimaging. All participants were supervised individually by experimenters outside and inside the scanner rooms. After a brief general explanation, they first filled out the standard MR-screening questionnaire and, if passed, they provided informed consent.

##### Penalty-Reading Task Outside the Scanner

Participants started the experiment by performing a practice session of the penalty-reading task, so they would be well prepared by the time they performed the same task inside the MR scanner.

##### Video-Training Outside the Scanner

Next, they started training with the video materials; half of them practiced first with the “attention” instructions and then with the “imagery” instructions’ the other half took the reverse order.

The attention training worked as follows. The participant watched the clips from penalty kicks (from CL or WC tournaments) and was instructed to try and learn to anticipate the direction in which the penalty shooter would kick, by discovering which features of the shooter’s movement are most predictive. They were instructed to attend to the length, angle, and speed of the run-up; the orientation of the torso, arms, and supporting leg during kicking; the degree and angle of moving the kicking leg, the orientation of the kicking foot, the side (inside or front) of the foot used for kicking, the side (lower or middle) of the ball where it is hit, and the like. They were asked to picture themselves being the goalkeeper, trying to “read” the shooter’s aim: which of these aspects of the shooter’s movements are best imagined in order to infer and predict the penalty direction?

The imagery training worked as follows. The participant again was instructed to try and learn to anticipate the direction in which the penalty shooter would kick, by imagining as vividly as possible what it feels like to take penalties in various directions, given the way the penalty shooter moves. They were instructed to imagine feeling the movement of their torso, arms, supporting leg, and kicking leg during the run-up and especially during kicking; to imagine feeling the strength and speed of the movement; and to imagine feeling their body posture, balance, muscle tension, adrenalin rush, breath, or heartrate. They were asked to picture themselves being the goalkeeper, trying to “read” the shooter’s aim: which of these aspects are best attended to in order to infer and predict the penalty direction?

During either video training, incidentally (after every fourth mini-block) a text *“press a button to continue”* would appear, upon which they should push any button as fast as possible. This was a phony task, since other than that the participant did not have to actually do anything (other than stick to instructions); the phony response merely allowed us to verify that the participant was still attentive.

Imaging was conducted with a Phillips 3T Intera MR scanner using a 32-channel SENSE head coil at the Spinoza Centre for Neuroimaging at the Amsterdam University Medical Center. For anatomical referencing, a high-resolution 6-min T1-weighted structural scan was acquired first for each participant (T1 turbo field echo, TR 8.2 s, TE 3.8 ms, 220 slices, slice thickness 1 mm, voxel size 1 × 1 × 1 mm, FOV 240 × 188 mm, flip angle 8°). During the Video-training and penalty-reading tasks inside the scanner, the blood oxygen dependent (BOLD) signal was measured with a T2*single shot echo planar imaging (EPI) sequence (TR 2.0 s, TE 27.6 ms, 37 slices, slice thickness 3 mm, voxel size 3 × 3 × 3 mm, interslice gap 0.3 mm, FOV 240 × 121 mm, flip angle 76.1°).

After the participant was positioned in the MR scanner, an anatomical scan of 10 min was taken.

##### Penalty-Reading Task and Video-Training Inside the Scanner

The video training and penalty-reading task were performed as described under *Materials*. Participants were asked explicitly not to move during the session, other than when asked to respond, and then only with the muscles pertinent to that response. Responses were issued by pressing the left and right index and middle finger to indicate the left and right bottom or upper corner respectively on hand-held scanner-compatible button-boxes.

#### Data Analysis

##### MRI Preprocessing

Results included in this manuscript come from preprocessing performed using *FMRIPREP* version 0.6.2 (Esteban et al., 2019), a *Nipype* (Gorgolewski et al., 2011) based tool. Each T1-weighted volume was corrected for bias field using *N4BiasFieldCorrection* v2.1.0 (Tustison et al., 2010) and skullstripped using *antsBrainExtraction.sh* v2.1.0 (using the OASIS template). The skullstripped T1-weighted volume was co-registered to skullstripped ICBM 152 Nonlinear Asymmetrical template version 2009c (Fonov et al., 2009) using nonlinear transformation implemented in *ANTs* v2.1.0 (Avants et al., 2008).

Functional data were motion-corrected using *MCFLIRT* v5.0.9 (Jenkinson et al., 2002). This was followed by co-registration to the corresponding T1-weighted volume using boundary-based registration 9 degrees of freedom—implemented in FSL (Greve and Fischl, 2009). Motion correcting transformations, T1 weighted transformation, and MNI template warp were applied in a single step using *antsApplyTransformations* v2.1.0 with Lanczos interpolation. The time series were subsequently high-pass filtered using FSL with a threshold of 100 s and each volume was spatially smoothed using FSL with an FWHM of 5 mm.

Three tissue classes were extracted from T1-weighted images using *FSL FAST* v5.0.9 (Zhang et al., 2001). Voxels from cerebrospinal fluid and white matter were used to create a mask which in turn is used to extract physiological noise regressors using aCompCor (Behzadi et al., 2007). Mask was eroded and limited to subcortical regions; to limit overlap with gray matter, six principal components were estimated. Frame-wise displacement (Power et al., 2014) was calculated for each functional run using Nipype implementation. For more details of the pipeline see https://fmriprep.readthedocs.io/en/0.6.2/workflows.html.

First-level (participant-specific) single-trial models were estimated using *FSL FEAT* (Woolrich et al., 2001). Each model contained separate predictors for each trial (lasting for the duration of the video) convolved with a double-gamma HRF, as well as time series from the six motion realignment parameters and a single global signal (i.e., average time series across all voxels) time series. The resulting whole-brain *z*-statistic maps of all trials (i.e., 60 per run) were subsequently used in the MVPA analyses.

##### Model Training

For each participant, a support-vector classifier with a linear kernel and default hyperparameters as implemented in the Python package *scikit-learn* (Pedregosa et al., 2011) was iteratively trained on 90% of the trials to distinguish the task condition: either attention (ATT) or imagery (IMG). The classifier was then evaluated on the remaining 10% of the ATT and IMG trials (i.e., 10-fold cross-validation). Because the data was balanced in terms of class frequency, model performance was summarized as the average accuracy across 10 folds. This average accuracy was statistically tested against chance level (i.e., an accuracy of 50%) using a permutation analysis (Ojala and Garriga, 2010) with 1,000 iterations in which the classifier was trained and evaluated on shuffled target labels.

Importantly, because the voxel time series may still contain low-frequency drift, trials from the same task may be temporally correlated, violating the independence assumption of cross-validation and thus likely yielding inflated accuracy scores. This issue is, notably, not present in the cross-classification analysis, because the trials from the two tasks (ATT/IMG and penalty-reading) are in fact independent.

To visualize the voxels that are most important in the classification analysis, we averaged (across folds) the classifier weights for each participant and computed for each voxel a two-sample *t*-test (against a population value of 0) of the fold-average classification weights across participants.

##### Model Cross-Classification

**Figure 3:**
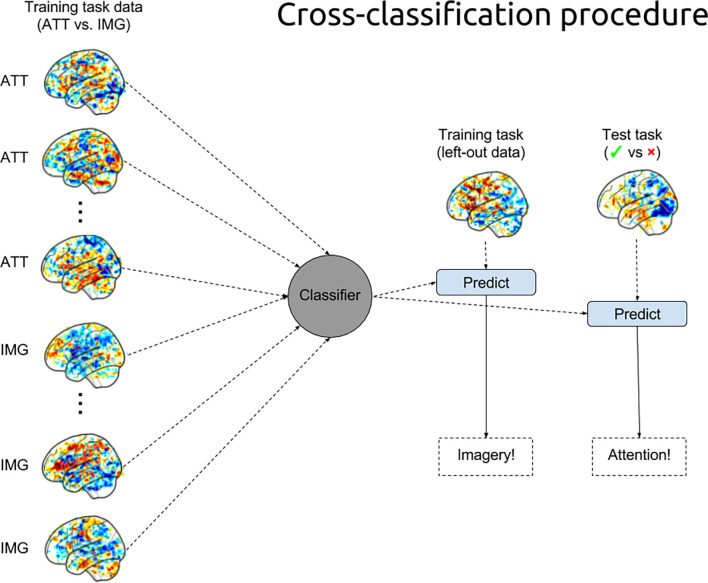
MVPA training and cross-classification procedures. The pattern classifier was trained on 90% of the data of each participant in the training task to distinguish attention (ATT) trials from imagery (IMG) trials and was used to predict (and evaluate) the remaining trials from the training task as well as to predict the trials from the test task (i.e., the cross-classification of the penalty-reading task).

For the cross-classification analysis, we evaluated the classifier (as specified in the previous section) trained on the ATT and IMG trials on the trials from the penalty-reading task, again for each participant separately. Thus, each trial in the penalty-reading task was classified as either ATT or IMG. See Figure 3 for a visualization of the training and cross-classification procedure.

We then computed, per participant, the proportion of ATT and IMG predictions for the “blocked” (i.e., accurately anticipated) trials from the penalty-reading task. This is equivalent to the classifier’s *recall* score when the correctly anticipated trials are regarded as the positive class. If the engagement of the motor imagery circuitry would contribute to successfully blocking a penalty, we would expect a relatively high proportion (i.e., >50%) of IMG predictions for these trials. Similar, to the permutation analysis described in the previous section, we ran a permutation analysis for the cross-classification models by training the models, for each participant separately, on shuffled target labels and evaluating them again on the trials from the penalty-reading task. Finally, statistical significance on the group-level statistics was computed using a two-sample *t*-test against 50% (chance level) of the proportion of IMG predictions for the successfully blocked penalty trials across participants.

### Results

#### Training Task

**Figure 4:**
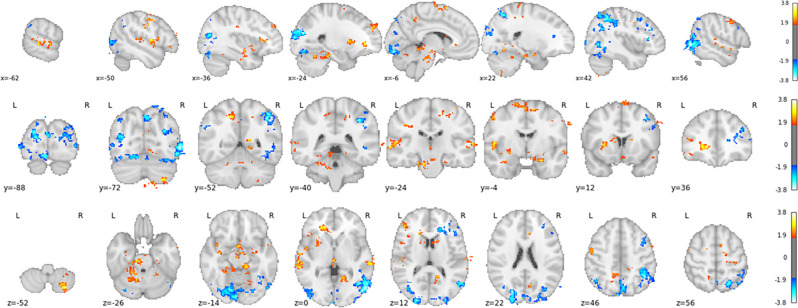
T-value map of classifier weights, computed using a one-sample *t*-test against 0, thresholded at *t* > 1.7 and only showing clusters with more than 200 voxels. Clusters in yellow/red and blue are associated with imagery and attention predictions, respectively.

The classifier was able to correctly classify the trials from the training task significantly above chance-level (i.e., 50%) for all participants (*p* < 0.001), with an average (across participants) accuracy score of 94% (*SD*: 0.045). However, as discussed previously, the magnitude of the results is likely inflated due to a likely violation of independence across trials from the same condition. Regardless, the spatial pattern of classifier weights (see Figure 4) show to-be expected regions associated with the attention condition (primarily occipital and parietal cortex) and the motor imagery condition (such as the supplementary motor complex, the caudate nucleus, and the cerebellum).

#### Penalty-Reading Task

The participant-specific analyses showed that the proportion of IMG predictions for the correctly anticipated trials was significantly higher than chance in twenty out of the total number of 35 participants (i.e., 57%; see also Supplementary Figure 1). At the group-level, in line with our expectations, the proportion of successfully blocked trials predicted as motor imagery was, on average across participants, 62.2% (*SD*: 0.246), which was significantly above chance (*t*_(34)_ = 2.925, *p* = 0.006).

### Discussion

Using MVPA, a classifier was trained to discern the circuits unique to motor imagery from the circuits involved in attention to kinematic features of penalties. When applied to the untrained subset of the data, the classifier correctly categorized the trials as belonging to the imagery or attention instructions at high accuracy. We then employed MVPA-CC and observed that individuals use their motor imagery circuitry in roughly two-thirds of all successfully read penalty kicks, which was significantly above chance. The predictive clusters in the motor-imagery circuitry corresponded roughly to at least some of the regions of the motor-imagery network depicted in Figure 1 (e.g., the supplementary motor complex, the caudate nucleus, and the cerebellum). Thus, we established that KMI is used (at least part of the time) to successfully anticipate the direction of the opponent’s penalty kick. Consistent with the predictions derived from IMPPACT, we conclude that pre-play in the form of KMI is used in reading other people’s body language to infer their action intention. By inference, we assume that reading others’ action intentions invokes “inverse modeling”, which requires the presence of a forward model that can be enriched using KMI.

A number of factors may potentially limit the straightforwardness of these conclusions. First, despite instructions and design features, we can’t know what participants are actually doing when viewing the training fragments. For instance, it may be possible that they distribute attention across all possible kinematic parameters in the attention condition, whereas they focused specifically on one parameter (such as the orientation of the supporting leg) in the KMI condition. Although such scenarios cannot be excluded, they seem unlikely, as (in this example) there should be a specific neural circuitry that is engaged specifically in focusing on the kinematic parameter of the orientation of the supporting leg, substantially more than on all other kinematic parameters together. While not impossible, we are not aware of data implying such specific sensitivity to specific motion parameters.

Second, there is no real way of knowing whether the participants actually moved muscles during motor imagery while keeping their heads still. If so, the neural activity related to the muscle contractions might be what the pattern classifier picked up on; in fact, this may have contributed to the relatively high accuracy of classification. However, this would not likely explain the successful cross-classification unless participants contracted the exact same muscles during the video training and the penalty anticipation task.

Third, in recent years we have seen a rapid increase in the preferred number of participants in fMRI research. While 35 was considered adequate when we initiated this study, this number may already be on the small side compared to present standards. Thus, it will be useful to try and replicate this study with a larger sample.

Forth, the design for the fMRI study in Experiment 3 was constructed such that the attention and imagery trials were grouped in separate fMRI runs. As mentioned, this design most likely induced temporal correlations across single trials within a particular condition, increasing their neural pattern similarity. This in turn might incur dependence across trials, which likely inflated accuracy scores for the model performance. To avoid this issue, future research should make sure that trials (or, equivalently, trial blocks) from different conditions are properly randomized across fMRI acquisition runs (for details, see Mumford et al., 2014). However, because the training and test task (i.e., the penalty-reading task) were independent, this issue of temporal correlations across trials is not applicable to the cross-classification analysis.

Notwithstanding these minor limitations, we believe that the current findings constitute reasonably strong evidence in favor of the notion that pre-play is used in reading other people’s body language to infer their action intention. The present demonstration further underlines the notion that MVPA and especially cross-classification comprises a sensitive tool to uncover the hidden mechanisms underlying complex cognitive processes represented in distributed cortical networks.

## General Discussion

Key to the coordination of our actions with those of others is the ability to “read” the actions of others and the intentions behind them. Based on predictive processing theory, we hypothesized that in order to read someone else’s action intention, one needs to have a rich kinesthetic experience with that action oneself. We applied this conjecture to the special case of penalty-reading. In a series of studies, we tested the nontrivial prediction that penalty-reading performance in soccer improves after practicing the kinematics and/or kinesthetics of penalty-*kicking*.

### Summary of Findings

In Experiment 1, we developed a direct test of the hypothesis that the more kinesthetic experience a goalkeeper has in penalty-*kicking*, the more effectively s/he can predict the shooter’s aim, thus improving her/his chances to prevent the shooter from scoring a goal. We observed that not only practicing in penalty-blocking but also practice in penalty-kicking improved penalty-reading accuracy, which cannot be explained by experiencing penalty-blocking situations* per se*. Future studies might include players that differ in level and years of experience to see if these moderate the observed effects.

In Experiment 2, we examined whether similar benefits can be obtained by motor imagery, that is, by vividly mimicking and experiencing the shooter’s movement in one’s mind. As it turned out, practicing kinesthetic motor imagery of penalties failed to improve penalty-reading accuracy, in either the penalty-blocking or the penalty-kicking condition, thus disconfirming our predictions. KMI of penalty kicking apparently failed to strengthen and enrich the forward model of penalty kicking enough for the observer to improve in reading an opponent’s penalty kick. A number of observations urged for caution in accepting this falsification as conclusive, however. First, our implementation of KMI might not have been sufficiently powerful; the fact that practice in KMI of penalty *blocking* also failed to produce improvements may suggest that our KMI sessions were not successful in instilling *any* effects, at least in the present samples. Possibly, for some individuals in the present experiment, the vividness of KMI was limited, which would also limit the chances of finding *any* effect of KMI practice. Second, both accuracy and response speed were considerably poorer among the novice participants with little experience in penalty kicking or blocking in Experiment 2 than in the experienced goalkeepers in Experiment 1. Stronger effects might be obtained by testing the KMI experiment with goalkeepers. Finally, perhaps KMI should build on physical training rather than being administered separately. A replication (with larger samples) might focus on combining physical and virtual training to examine if and when KMI might contribute to performance beyond physical training.

The finding that practice in KMI of penalty-kicking and penalty-blocking did not improve penalty-reading skilsl does not exclude the possibility that KMI *is* used during penalty reading in the first place. This possibility was tested in Experiment 3, in which we trained a machine-learning pattern classifier on fMRI data to test (using MVPA-cross-classification) whether motor-imagery brain networks are engaged in successful penalty reading. We observed that individuals use their motor imagery circuitry in roughly two-thirds of all successfully “read” penalty kicks. Thus, KMI was used (at least part of the time) to successfully anticipate the direction of the opponent’s penalty kick. Although the fMRI design likely induced temporal correlations across single trials within a particular condition, which may have inflated accuracy scores for the model performance, this issue of temporal correlations across trials is not applicable to the cross-classification analysis, since the training and test task (i.e., the penalty-reading task) were independent. The findings suggest that pre-play is used in reading other people’s body language to infer their action intention.

### Implications

The results from Experiment 1 revealed that, after merely 20 min of training, practice in penalty-kicking improved the accuracy of penalty-reading by 4.5%. Given that professional goalkeepers in the German *Bundesliga* block 18.8% of all penalty kicks (Dohmen, 2008), an increase of 4.5% would be massive, and may well imply the difference between winning and losing a match (or a tournament, for that matter). Although the goalkeepers tested in this experiment were experienced high-level amateurs, it remains to be established, of course, whether the improvements extend beyond the experimental setting and if professionals in national soccer competitions also benefit as much. Yet, this is a most encouraging result, opening the stage for expanding goalkeeper training strategies to increased experience in penalty-kicking. Responding after the penalty ball has been hit leaves the goalkeeper with too little time to arrive before the ball crosses the goal line (Glencross and Cibich, 1977; Chiappori et al., 2002); reading the shooter’s movements during the run-up and during the kick may give the goalkeeper a head-start and an increase in their probability of blocking the penalty.

Improved performance by attempting to “read” the penalty through an assessment of the shooter’s kinematic body and movement parameters is consistent with the predictions derived from IMPPACT (Ridderinkhof, 2014) that build on the notion of forward modeling, which is key to modern theories of active inference and predictive processing (e.g., Wolpert et al., 2003; Friston et al., 2011; Clark, 2013). Note that the central notion of deciphering others’ action intentions was formulated already 100 years ago by Edward Kempf: *“understanding the behavior of others — that is, by miniature tonal forms of reflex reproduction of the movements of others — the proprioceptors, giving the appropriate kinesthetic sensations, enable the personality to become aware of the significance of the posture and movements or behavior of others”* (Kempf, 1921, p.22). This skill of “reading” action intentions is of obvious evolutionary-adaptive value to social animals: in fighting, courting, and all kinds of joint and complementary action, animals need to be able to read other animals’ body language. The goalkeeper needs to infer, based on observations of the kicker’s run-up and shooting kinematics, the orientation of the supporting leg, etc., the intention of the shooter (which angle will s/he take), and then act accordingly (cf. Kilner et al., 2007; Ridderinkhof, 2017). The present results are consistent with the novel hypothesis that for goalkeepers to read the body language of penalty kicks, they should be experts in kicking penalties themselves. The more experience a goalkeeper has in penalty-kicking, the more effectively s/he can inverse model and predict the shooter’s aim, thus improving her/his chances to prevent the shooter from scoring a goal.

The present data did not support the notion, building on the above conjecture, that kinesthetic motor imagery of penalty-kicking might suffice to bring about an improvement in penalty-reading. Neural activation observed during KMI has been found to display a reasonable correspondence with activation during the preparatory planning phase that precedes movement (Jeannerod, 2006), but also goes beyond mere preparatory planning, as demonstrated by the finding that KMI engendered activation in the contralateral primary motor cortex just as actual movements did (Stinear et al., 2006). Still, our instantiation of KMI lacked the power to induce a training benefit, at least in novices. Combining physical and KMI practice may perhaps result in more optimal benefits.

Although practice in KMI of penalty-kicking was not found to help improve penalty-reading skills, this does not imply that KMI is not used at all during penalty reading in the first place. Based on an MVPA-cross-classification procedure, we could demonstrate that in fact pre-play in the form of KMI was used (at least part of the time) for reading the opponent’s body language to infer their action intention and successfully anticipate the direction of the opponent’s penalty kick. By inference, we may speculate that reading others’ action intentions invokes inversing the forward model, which requires the presence of a rich forward model in the first place.

### In Conclusion

The key to action control is one’s ability to adequately predict the consequences of one’s actions. Reading another’s action intentions requires a rich forward model of that agent’s action; we showed that goalkeepers who had extensive prior experience in penalty blocking but not in penalty kicking can enrich their forward model of penalty kicking and use that to predict the direction of an imminent penalty kick. MVPA-cross-classification showed that 2/3 of all correctly read penalty kicks were classified as specifically engaging the circuitry involved in motor imagery of penalty kicking. In sum, this study provides initial evidence that it takes practice as a penalty kicker to become a penalty killer.

## Data Availability Statement

The raw data supporting the conclusions of this article will be made available by the authors, without undue reservation.

## Ethics Statement

The studies involving human participants were reviewed and approved by Ethics Review Board of the University of Amsterdam, Faculty of Societal and Behavioral Sciences. The patients/participants provided their written informed consent to participate in this study.

## Author Contributions

KR and AC performed the experiments. KR performed the data analysis of experiments 1 and 2. LS performed the data analysis of experiment 3. JC and AC developed scanning procedures for experiment 3. GS and AC prepared the video materials for the penalty-reading task. KR, LS, JC, GS, and AC wrote the text of the manuscript. All authors contributed to the article and approved the submitted version.

## Conflict of Interest

The authors declare that the research was conducted in the absence of any commercial or financial relationships that could be construed as a potential conflict of interest.

## Publisher’s Note

All claims expressed in this article are solely those of the authors and do not necessarily represent those of their affiliated organizations, or those of the publisher, the editors and the reviewers. Any product that may be evaluated in this article, or claim that may be made by its manufacturer, is not guaranteed or endorsed by the publisher.

## Supplementary Materials

Supplementary Figure 1 Observed scores (red vertical line) and distribution of permuted scores (blue histogram) for each participant-specific IMG/ATT model cross-validated to the trials from the penalty-reading task. Scores (x-axis) represent the proportion of correctly anticipated trials predicted as IMG (or, equivalently, the *recall* score).
