# Supplementary figures and images for: Action Intentions, Predictive Processing, and Mind Reading: Turning Goalkeepers Into Penalty Killers
Source: Front Hum Neurosci. 2022 Jan 20;15:789817. doi: 10.3389/fnhum.2021.789817 (PMC8812381; doi:10.3389/fnhum.2021.789817)

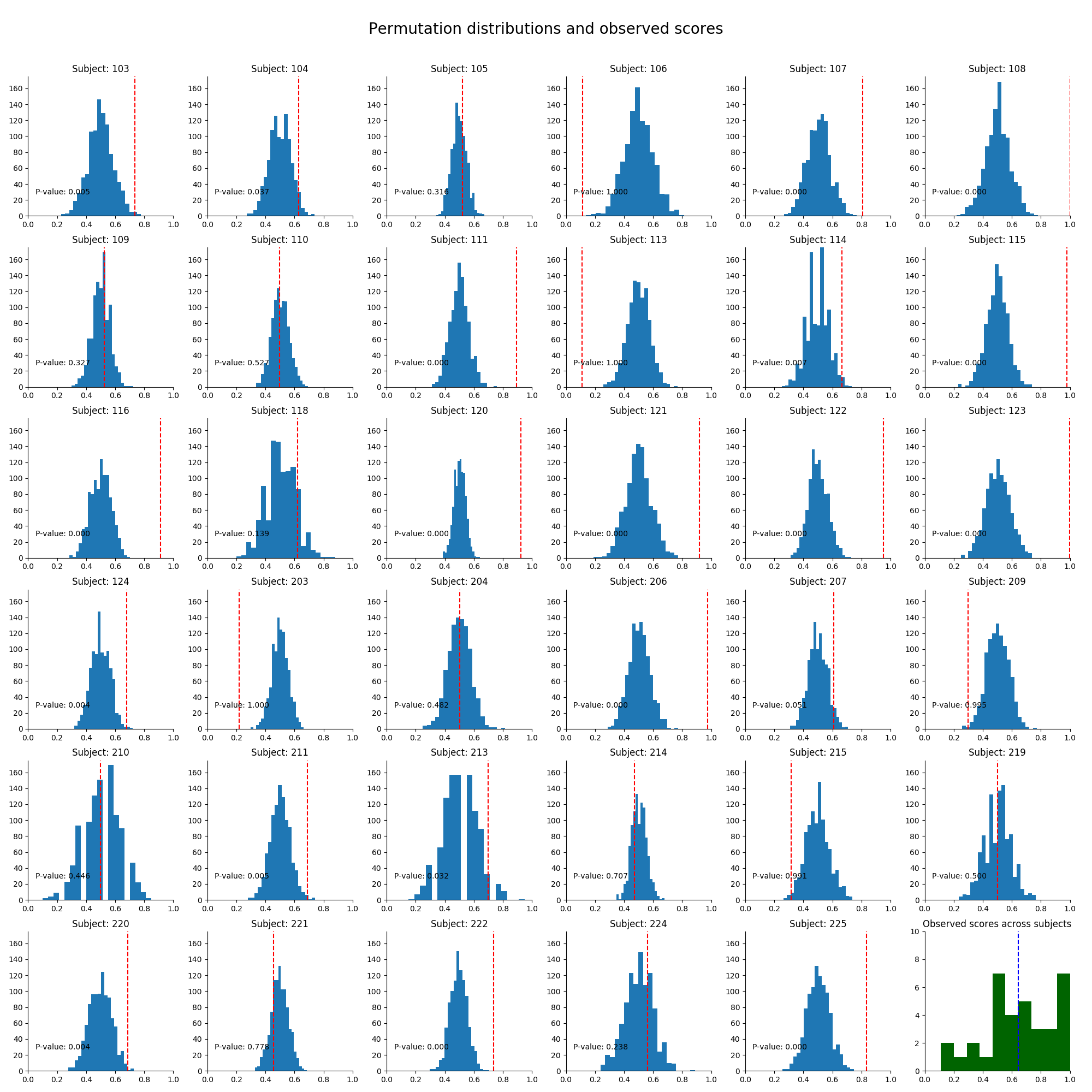

Supplement: Supplementary Figure 1 — Observed scores (red vertical line) and distribution of permuted scores (blue histogram) for each participant-specific IMG/ATT model cross-validated to the trials from the penalty-reading task. Scores (x-axis) represent the proportion of correctly anticipated trials predicted as IMG (or, equivalently, the recall score). [file Image_1.TIF]
